# Pediatric Alert Score: evaluation of measurement properties

**DOI:** 10.31744/einstein_journal/2025AO1214

**Published:** 2025-05-30

**Authors:** Vitória Yukari Chaves Aoki, Giselli Cristina Villela Bueno, Flávia Carvalho Pena Dias, Renata Cristina Gasparino, Daniela Fernanda dos Santos Alves

**Affiliations:** 1 Universidade Estadual de Campinas Faculdade de Enfermagem Campinas SP Brazil Faculdade de Enfermagem, Universidade Estadual de Campinas, Campinas, SP, Brazil.

**Keywords:** Pediatric nursing, Clinical deterioration, Early Warning Score, Child, hospitalized, Validation study, Reproducibility of results

## Abstract

**Objective:**

To evaluate the construct validity and reliability of the Pediatric Alert Score in hospitalized children and adolescents.

**Methods:**

This methodological study included 84 children aged 0-14 years. Data were collected using three instruments: a sociodemographic and clinical characterization form, the Brazilian version Brighton Pediatric Early Warning Score, and the Pediatric Alert Score. The reliability of the Pediatric Alert Score was assessed using interobserver equivalence expressed by the intraclass correlation coefficient, and the construct validity was evaluated using Spearman's correlation analysis of the Pediatric Alert Score and the Brighton Pediatric Early Warning Score.

**Results:**

The reliability analysis of the Pediatric Alert Score revealed an intraclass correlation coefficient of 0.95 (95% confidence interval from 0.93-0.97), and the Spearman's correlation coefficient was 0.53 (p<0.0001), indicating a strong positive correlation between the instruments.

**Conclusion:**

The Pediatric Alert Score demonstrated construct validity and internal consistency in the studied sample and can be used to enhance patient safety.

## In Brief

This study evaluated using the Pediatric Alert Score for hospitalized children aged 0-14. The Pediatric Alert Score demonstrated construct validity and internal consistency for the studied sample and can be used to enhance patient safety.

## Highlights

Clinical deterioration is a global concern related to patient safety.

The Pediatric Alert Score includes assessments of neurological, respiratory, and cardiovascular conditions; temperature; and urine output.

The Pediatric Alert Score demonstrated high reliability and strong validity with the B-PEWS-Br.

The Pediatric Alert Score is a reliable tool for detecting clinical deterioration and improving patient safety.

## Graphical abstract

**Figure:**
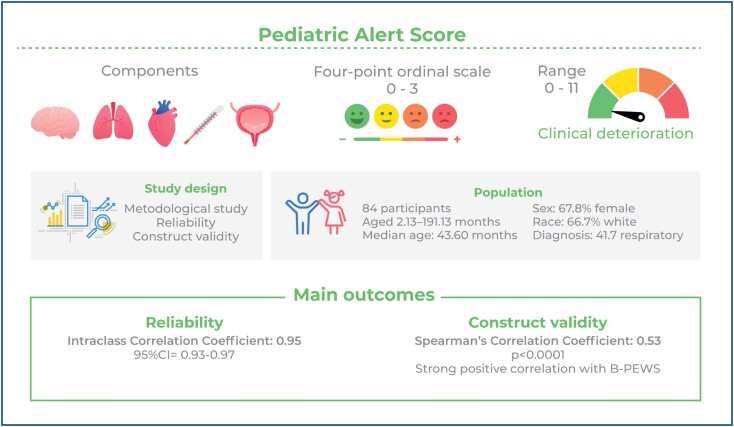

## INTRODUCTION

Clinical deterioration is a global concern related to patient safety and an important indicator of healthcare quality.^(1)^ Failures in the early identification of clinical changes can compromise patient survival and prognosis,^(2,3)^ which may be exacerbated by incorrect interpretation, record-keeping errors in medical charts, and even a lack of confidence in the observed vital signs.^(4,5)^ This gap in identifying warning signs may be related to the complexity of the environment, work overload, and weaknesses in communication processes among healthcare professionals, particularly between nurses and doctors.^(4)^

The nursing team plays a fundamental role in identifying, recording, and communicating the signs of clinical deterioration, thereby enabling decision-making in patient care. This process is continuous and includes observing and interpreting signs, symptoms, and patient behaviors to identify potential health and safety threats.^(5)^

Standardized early warning scores can assist in identifying warning signs and implementing early and timely treatment.^(3,5)^ These tools aim to facilitate the identification of clinical signs and symptoms through the assessment of healthcare professionals, triggering rapid actions after "trigger events," and avoiding unfavorable outcomes such as shock, respiratory failure, cardiopulmonary arrest, and death.^(3,4)^

Several instruments have been developed and validated for this purpose in pediatrics. Among them, the Brighton Pediatric Early Warning Score (B-PEWS) is widely applied, owing to its simplicity and ease of understanding, and can be used in children and adolescents up to 16 years of age.^(6)^ Brighton Pediatric Early Warning Score was developed by Monaghan in 2005 at the University of Brighton, England, with the participation of multidisciplinary team members who shared their assessment strategies regarding children's clinical conditions and disease severity levels. Since its creation, the B-PEWS has been applied in pediatric healthcare settings in the United States,^(7-12)^ Australia,^(13)^ Norway,^(14)^ the Netherlands,^(15)^ Argentina,^(16)^ and Brazil.^(17-19)^

In England and other countries, various models are used by different healthcare institutions to evaluate signs of clinical deterioration, suggesting the need for the implementation of a standardized system. Despite the divergence in the adopted alert scores, they have more similarities than differences, which facilitates the transition to a single system.^(20)^

The Brazilian version of the B-PEWS (B-PEWS-Br) has been adapted and validated according to international standards, showing evidence of its validity and reliability.^(17,19)^ Validating instruments according to the population served is crucial, as evidenced by a Dutch study highlighting the implementation of a pediatric early warning system based on B-PEWS. The lack of a validated instrument according to Dutch guidelines resulted in the adoption of modified and unvalidated scores in some hospitals, creating a false sense of security and increasing the risk of clinical deterioration signs being overlooked by professionals.^(21)^

The application of the B-PEWS-Br in hospitalized children has been validated by several nationwide studies.^(17-19,22)^ It is an easy-to-apply tool that requires few resources for implementation and can be incorporated into the daily routines of inpatient units with greater applicability. Additionally, the staff of inpatient units are less accustomed to the main signs of sudden clinical complications, unlike the staff of critical care units, where urgent and emergency situations are more frequent.^(12-17)^ Thus, the early detection of clinical deterioration becomes especially important in hospitals with limited resources, as preventing clinical worsening reduces the need for transfer to intensive care units and consequently helps decrease costs related to human resources, materials, and other technologies.^(23)^

Recently, a new instrument for assessing pediatric clinical deterioration tailored to the national context, the Pediatric Alert Score (PAS), was developed.^(24)^ The PAS was created based on the B-PEWS-Br. The discussion on the PAS began after the adaptation and validation of the B-PEWS-Br, owing to the need to improve the instrument in the national context and include important clinical indicators for the identification of sepsis and/or septic shock. Therefore, in addition to the neurological, respiratory, and cardiovascular assessments of the B-PEWS-Br, two new components were introduced: temperature and urine output.^(24)^

The PAS has been described as a modified version of the B-PEWS-Br, and its development included collaboration with the author of the B-PEWS.^(24)^ Importantly, although the B-PEWS-Br has proven to be a valid instrument, the authors emphasize the need to validate new tools, since to date, only the B-PEWS-Br has been validated in the Brazil.^(17)^

The hospital unit chosen for the study does not employ an early warning system, although its implementation in the electronic patient information management system is planned. Therefore, providing a tool with proven construct validity and internal consistency can aid in planning the necessary human and technological resources to implement the instrument in the study unit.

## OBJECTIVE

To evaluate the construct validity and reliability of the Pediatric Alert Score in hospitalized children and adolescents.

## METHODS

### Study design

This methodological study followed the recommendations for validation studies published by the COnsensus-based Standards for the selection of health Measurement INstruments (COSMIN).^(25)^

### Data collection site

This study was conducted in the pediatric inpatient unit of a public teaching hospital in the interior of São Paulo, Brazil. The institution has 42 beds for children and adolescents with medium-to high-complexity conditions. Additionally, the 405-bed teaching hospital provides tertiary care and caters to patients with high-complexity conditions.

This in-person study was conducted February 2022 to March 2023.

### Participants and selection criteria

The sample size was determined using the recommended hypothesis testing criteria for reliability and construct validation studies, in which a minimum "adequate" sample size is 50 patients.^(25)^ For construct validation, a test power of 80%, significance level of 5%, estimated correlation coefficient of 0.30, and null hypothesis correlation coefficient of 0.00 were used to calculate a minimum sample size of 84 participants.^(26)^ Participants were selected non-probabilistically and consecutively. The inclusion criteria were children or adolescents aged 0-14 years, hospitalized for more than 24 h for any clinical condition, and accompanied by parents or guardians. Children and adolescents diagnosed with heart disease were excluded, to ensure sample homogeneity.

### Instruments

#### Sociodemographic and clinical characterization form

Sociodemographic and clinical variables were selected based on previous studies^(18,19)^ and included age, sex, family income, inclusion in a social program, diagnosis, comorbidities and previous hospitalization,

#### Brighton Pediatric Early Warning Score - Brazilian Version (B-PEWS-Br)^(17)^

The B-PEWS-Br consists of three assessment components: neurological, cardiovascular, and respiratory. The neurological status evaluates the child's level of activity; the cardiovascular component assesses the skin color, heart rate (HR), and capillary refill time (CRT); and the respiratory component evaluates the respiratory rate (RR), use of accessory muscles, and oxygen support. Each component was assessed on a four-point ordinal scale, from zero to three, and the total score ranged from 0 to 13 points, with higher values reflecting greater clinical deterioration. Two additional points were added when the child or adolescent had undergone nebulization or experienced vomiting after surgery. Scores ≥3 indicated signs of clinical deterioration and required intervention by the healthcare team.^(17-19)^ The interobserver agreement index obtained in a previous application resulted in a kappa of 0.85 (95% confidence interval [95%CI]: 0.55-1.00),^(19)^ with a sensitivity of 73.9% and a specificity of 95.5%.^(17)^

### Pediatric Alert Score (PAS)^(24)^

The PAS consists of neurological, respiratory, and cardiovascular assessments and two additional components: temperature and urine output. Each component was assessed on a four-point ordinal scale, from zero to three, with higher values reflecting greater clinical deterioration; total scores ranged from 0 to 11 points.^(24)^ The urine output and temperature were considered relevant clinical signs for cardiovascular assessment, and they were added with the approval of the instrument development committee. Urine output is a measure of target organ perfusion, and a reduced or absent urine volume may indicate decreased renal perfusion. Temperature is an important indicator; hypothermia is a preventable cause of cardiopulmonary arrest, and hyperthermia can be associated with infections, which, if untreated, may progress to sepsis. The PAS layout features columns that are color-coded according to the severity of the indicator.^(17,24)^

### Data collection

Data was collected in person, and the three instruments were integrated into the Research Electronic Data Capture (REDCap) platform to ensure data quality. Data were de-identified, to preserve participant confidentiality.

In the first stage of data collection, one researcher reviewed the daily census of each unit and applied the eligibility criteria. Subsequently, the parents and/or guardians of potential participants were invited to participate and informed of the research objectives, risks, and benefits. All parents and guardians signed the informed consent form (ICF). Children and adolescents older than seven years received explanations about the study and signed the assent form (AF). After obtaining the signatures, the same researcher collected sociodemographic and clinical information through interviews with the parents and/or guardians. Missing information was obtained by reviewing electronic medical records.

In the second stage, to assess clinical signs of deterioration, two researchers independently and simultaneously applied the PAS. Only one researcher applied the B-PEWS-Br.

### Data analysis

The analysis was performed using the Statistical Analysis System (SAS) for Windows (version 9.4) and the SPSS; version 23. Descriptive statistics were used to describe the participants’ profiles and calculate the mean, median, standard deviation, and minimum and maximum values for continuous variables. Categorical variables are presented as the numbers (n) and frequencies (%). The Shapiro-Wilk test was used to evaluate the data distribution. The reliability of the PAS was assessed using interobserver equivalence, expressed using the intraclass correlation coefficient (ICC),^(27)^ and values >0.70 indicated good reliability.^(25)^

The hypothesis that a higher PAS score would correlate with a higher B-PEWS-Br score was tested using the construct validity and the Spearman's correlation coefficient.^(28)^ This coefficient ranged from −1 to 1, where values of 0.1-0.29 indicated a weak correlation, 0.30-0.49 suggested a moderate correlation, and ≥0.50 indicated a strong correlation.^(29)^

### Ethical aspects

The project was approved by the *Universidade Estadual de Campinas* Research Ethics Committee (CAAE: 53153221.2.0000.5404; #5.217.629) and adhered to the ethical recommendations for research involving humans according to Resolution 466/2012 of the National Health Council.

## RESULTS

### Participant profile

The clinical and sociodemographic profiles of the study participants (n=84) are presented in table 1. Participants were aged 2.13-191.13 months (median: 43.60 months; interquartile range [IQR]: 10.47-106.77 months). Most of the participants were female and white, had a family income between one and two minimum wages, and did not participate in any social assistance program. Regarding the clinical profile, the most frequent diagnoses were related to respiratory system problems, and more than half of the participants had a history of comorbidities, although 58 (69.0%) participants had no history of hospitalization.

**Table 1:** Sociodemographic and clinical characteristics of children and adolescents

Variables		n (%)
Sex		
	Female	57 (67.8)
	Male	27 (32.2)
Race		
	White	56 (66.7)
	Mixed	21 (25.0)
	Black	7 (8.3)
Family income†		
	<1 minimum wage	29 (34.5)
	1 to <2 minimum wages	41 (48.8)
	2 to 4 minimum wages	12 (14.3)
	>4 minimum wages	1 (1.2)
	No information	1 (1.2)
Type of social assistance		
	None	54 (64.3)
	Brazil Benefit	8 (9.5)
	INSS Benefit‡	3 (3.6)
	Family Benefit	4 (4.8)
	Other	6 (7.1)
	No information	9 (12.0)
Diagnoses by system		
	Respiratory	35 (41.7)
	Renal	10 (11.9)
	Gastrointestinal	11 (13.1)
	Neurological	7 (8.3)
	Surgical indications	6 (7.1)
	Endocrine and metabolic	4 (4.8)
	Musculoskeletal	3 (3.6)
	Hematological	2 (2.4)
	Other	6 (7.1)
Presence of comorbidities		
	Yes	49 (58.3)
	No	35 (41.7)
Number of comorbidities		
	None	36 (42.9)
	1-2	30 (35.7)
	3-4	13 (15.5)
	≥5 or more	5 (6.0)
Previous hospitalization		
	No	58 (69.0)
	Yes	26 (31.0)

† Family Income = Minimum Wage in 2022 (R$ 1,212.00) and in 2023 (R$ 1,320.00) in Brazil;

‡ INSS = National Institute of Social Security.

### Reliability and validity of the PAS

The reliability index of the PAS showed an ICC of 0.95 (95%CI= 0.93-0.97) (Table 2). For both observers, the mean score was 1.60 for both observers (minimum: 0; maximum 8).

**Table 2:** Distribution of interobserver reliability measures for the Pediatric Alert Score

Variable	n	Mean	SD	Minimum	Q1	Median	Q3	Maximum	ICC	95% CI
Observer 1	84	1.60	1.84	0.00	0.00	1.00	2.00	8.00	0.95	0.93-0.97
Observer 2	84	1.60	1.99	0.00	0.00	1.00	2.00	8.00		

When the B-PEWS-Br and PAS were applied, 26.67% and 24.0% of participants had scores ≥3, respectively. The construct validity of the PAS, expressed using the Spearman correlation coefficient, was 0.53 (p<0.0001), indicating a strong positive correlation between the instruments.^(29)^

## DISCUSSION

Clinical deterioration is the progressive worsening of a patient's physiological condition, which can cause preventable harm when recognized late.^(20)^ Recognizing clinical deterioration in pediatric patients is very challenging, mainly due to characteristics inherent to this population, such as difficulty in communicating symptoms as effectively as adults, a tendency to physiologically compensate (hindering symptom recognition), and rapid decompensation. However, the use of scores can enable communication among healthcare professionals and improve the quality of patient chart documentation.^(16)^

One in every five pediatric deaths in a hospital setting is estimated to involve preventable factors.^(20)^ Thus, the early detection of these signs can contribute to the quality of care by facilitating timely recognition before the patient requires transfer to the intensive care unit or emergency procedures.^(16)^

Globally, more than 30 instruments for identifying signs of pediatric deterioration, which have undergone different validation processes, have been developed.^(20)^An analysis of the use of these instruments in England concluded that despite differences, most instruments had the same "basic indicators" (*i.e*., assessment of neurological, cardiovascular, and respiratory components).^(20)^ However, the choice of one of these instruments must consider the validation processes of the scores. The implementation of one of these tools should be preceded by adaptation and validation processes for the desired context, as there is a wide variety of instruments and little proven evidence of their clinical utility.^(24)^

Thus, evaluating constructs using rigorously validated instruments is essential to ensure reliable and safe tools for clinical practice and research. Evidence suggests that validity, reliability, and responsiveness are important properties for assessing the quality of measurement instruments. Using more than one measure is recommended, and independent and complementary properties were followed in the present study.^(25,28)^

Reliability is the ability to produce consistent results when applied under the same conditions;^(25,28)^ in this study, the ICC demonstrated that the results obtained with the PAS are reliable for clinical practice. During the application, the researchers did not encounter difficulties with the instrument, which was easy to understand and interpret.

Validity, a characteristic of the instrument that determines whether it measures exactly what it proposes,^(25,28)^ was analyzed through hypothesis testing with the B-PEWS-Br. The results demonstrated that the PAS measures the clinical deterioration of pediatric patients, as supported by the positive, significant, and strong correlation coefficient. No studies have examined the use of the PAS beyond the location where it was developed.^(24)^ Thus, the data presented in this study may encourage further studies on the use and impact of scores in a national context.

The percentage of patients with scores indicative of clinical deterioration was slightly different between the two instruments; however, overall, they suggested that one in four children admitted to pediatric units showed warning signs. One possible cause for this difference is the indicators in each tool. The B-PEWS-Br retained the indicators from the B-PEWS: "nebulization within the last 15 minutes" and "persistent vomiting after surgery," each of which was scored 2 points if present. For the PAS, the authors excluded these indicators and added other assessment components. The majority of the study population consisted of children diagnosed with respiratory problems (*i.e*., requiring supplemental oxygen or nebulization), which may have influenced the results.

Assessing the predictive ability of the PAS was not possible, as <10% of patients developed hemodynamic instability, or required urgent interventions or transfer to critical care units (unpublished data), which may indicate a methodological limitation of this study.

Notably, the use of early warning scores does not have predictive power in isolation. Training and educating healthcare professionals regarding the use of the instrument, as well as implementing active learning strategies such as clinical simulation, can contribute to the early identification of signs of clinical deterioration.^(20)^ Furthermore, both the clinical judgment of the professional and the concerns of parents and caregivers should be considered paramount to any score, when appropriate.

Importantly, the instrument only allows for the early detection of clinical signs; therefore, before implementation, it is necessary to verify that the institution has adequate resources for standardizing rapid response protocols, considering the entire flow, from the moment of trigger to the activation of response procedures, for effective action regarding the patient.

According to the data obtained in this study, the PAS can be implemented nationally. At the location where this research was conducted, the results will be considered when choosing the tool to be implemented in the pediatric unit, which will be operationalized through the patient's electronic medical records.

## CONCLUSION

In the study sample, the Pediatric Alert Score demonstrated construct validity and reliability and can be used to detect clinical deterioration in hospitalized children and adolescents.
